# Comparing conVEntional RadioTherapy with stereotactIC body radiotherapy in patients with spinAL metastases: study protocol for an randomized controlled trial following the cohort multiple randomized controlled trial design

**DOI:** 10.1186/s12885-016-2947-0

**Published:** 2016-11-21

**Authors:** Joanne M. van der Velden, Helena M. Verkooijen, Enrica Seravalli, Jochem Hes, A. Sophie Gerlich, Nicolien Kasperts, Wietse S. C. Eppinga, Jorrit-Jan Verlaan, Marco van Vulpen

**Affiliations:** 1 Department of Radiation Oncology, University Medical Center Utrecht, Heidelberglaan 100, 3584 CX, Utrecht, The Netherlands; 2 Julius Center for Health Sciences and Primary Care, University Medical Center Utrecht, Utrecht, The Netherlands; 3 Department of Orthopedic Surgery, University Medical Center Utrecht, Utrecht, The Netherlands

**Keywords:** VERTICAL trial, Randomized controlled trial, Cohort multiple Randomized Controlled Trial design, Spinal metastases, Bone metastases, Pain, Stereotactic body radiotherapy

## Abstract

**Background:**

Standard radiotherapy is the treatment of first choice in patients with symptomatic spinal metastases, but is only moderately effective. Stereotactic body radiation therapy is increasingly used to treat spinal metastases, without randomized evidence of superiority over standard radiotherapy. The VERTICAL study aims to quantify the effect of stereotactic radiation therapy in patients with metastatic spinal disease.

**Methods/design:**

This study follows the ‘cohort multiple Randomized Controlled Trial’ design. The VERTICAL study is conducted within the PRESENT cohort. In PRESENT, all patients with bone metastases referred for radiation therapy are enrolled. For each patient, clinical and patient-reported outcomes are captured at baseline and at regular intervals during follow-up. In addition, patients give informed consent to be offered experimental interventions. Within PRESENT, 110 patients are identified as a sub cohort of eligible patients (i.e. patients with unirradiated painful, mechanically stable spinal metastases who are able to undergo stereotactic radiation therapy). After a protocol amendment, also patients with non-spinal bony metastases are eligible. From the sub cohort, a random selection of patients is offered stereotactic radiation therapy (*n* = 55), which patients may accept or refuse. Only patients accepting stereotactic radiation therapy sign informed consent for the VERTICAL trial. Non-selected patients (*n* = 55) receive standard radiotherapy, and are not aware of them serving as controls. Primary endpoint is pain response after three months. Data will be analyzed by intention to treat, complemented by instrumental variable analysis in case of substantial refusal of the stereotactic radiation therapy in the intervention arm.

**Discussion:**

This study is designed to quantify the treatment response after (stereotactic) radiation therapy in patients with symptomatic spinal metastases. This is the first randomized study in palliative care following the cohort multiple Randomized Controlled Trial design. This design addresses common difficulties associated with classic pragmatic randomized controlled trials, such as disappointment bias in patients allocated to the control arm, slow recruitment, and poor generalizability.

**Trial registration:**

The Netherlands Trials Register number NL49316.041.14. ClinicalTrials.gov registration number NCT02364115. Date of trial registration February 1, 2015.

## Background

Bone metastases are a frequent distant manifestation of cancer, with the spinal column being the most common site [1]. Spinal metastases can induce cancer-related pain, mechanical instability, and neural compression, thereby causing morbidity and impacting on quality of life (QOL). Treatment is aimed at pain relief and prevention of neurological deficits. The treatment for most patients with symptomatic spinal metastases is standard external beam radiotherapy [2], which is moderately effective: around 60% of patients who undergo external beam radiotherapy experience pain relief [3]. Furthermore, pain relief is often incomplete with complete pain response rates ranging from 0 and 23% [3] and one in five patients needs re-irradiation [4]. Escalating the dose to the metastatic site might improve the pain response and prolong the duration of pain relief [5]. Dose escalation to spinal tumors using standard radiotherapy is complicated by the low tolerance of the spinal cord to radiation. Stereotactic body radiotherapy (SBRT) is able to deliver precise high-dose radiation to spinal metastases in single or multiple fractions, while sparing surrounding healthy tissues. Phase I and II studies have suggested that, for selected groups of patients, SBRT for spinal metastases may be accurate, safe, and effective [5, 6], with complete pain response in 54% of patients six months after SBRT [7]. Other authors even reported overall pain response rates around 90% [8–10]. To date however, no randomized controlled studies have been performed so equipoise still exist on the effectiveness of SBRT in comparison to standard radiotherapy. Therefore, we designed a pragmatic randomized controlled trial to compare conVEntional RadioTherapy with stereotactIC body radiotherapy in patients with spinAL metastases (VERTICAL) following the CONSORT statement [11].

## Methods/design

### Study design

This study is conducted within the Prospective Evaluation of interventional StudiEs on boNe meTastases (PRESENT) cohort [12]. All patients with bone metastases referred to the departments or radiation oncology or orthopedic surgery of the University Medical Center Utrecht are asked to participate in this prospective, observational cohort. Baseline and follow-up data are collected from clinical files, and patient-reported outcomes (PROMs, i.e. a pain inventory and QOL questionnaires) are collected at fixed time intervals. This study follows the cohort multiple randomized controlled trial (cmRCT) design as described by Relton and colleagues [13].

### Patient recruitment

At enrollment, patients give informed consent for collection of clinical and survival data, and can opt-in to provide PROMs. In addition, in a separate question, we ask patients for their broad consent for future randomization in trials that will investigate the effectiveness of experimental treatments [14]. Patients within the PRESENT cohort who meet the VERTICAL inclusion criteria (Table 1) are identified as a sub cohort of eligible patients. Eligible patients are PROMs-providing participants of the PRESENT cohort, have untreated symptomatic spinal metastases, and have given consent for broad randomization to experimental interventions. Patients are excluded if they are not able to undergo SBRT, have severe or progressive neurological deficits, received previous radiotherapy or surgery to the index site(s), or have a life expectancy less than three months. After a protocol amendment on September 23, 2015 to adjust to developments in clinical practice, also patients with non-spinal bony metastases are eligible.

**Table 1:** Selection criteria for the VERTICAL study

Inclusion criteria	Exclusion criteria
Participant in PRESENT cohort	Lesion in C1, and C2
Filling out PRESENT-questionnaires	Contraindication for MRI if MRI is indicated
Broad consent for randomization to experimental interventions	Radiosensitive histology such as multiple myeloma
Histologic proof of malignancy	Unable to undergo SBRT treatment
Imaging evidence of bone metastases	Patient with < 3 months life expectancy
For spinal lesions, per lesion no more than 3 consecutive spine segments involved with one unaffected vertebral body above and below	Chemotherapy or systemic radionuclide delivery within 24 h before and after SBRT
No more than 2 painful lesions needing treatment	Previous EBRT or SBRT to same level
For spinal lesions, no compression of spinal cord	For spinal lesions, unstable spine requiring surgical stabilization
No or mild neurological signs^a^	Severe, worsening or progressive neurological deficit
KPS > 50 and pain score > 3^b^	

*VERTICAL* randomized controlled trial comparing conVEntional RadioTherapy with stereotactIC body radiotherapy in patients with spinAL metastases; *PRESENT* Prospective Evaluation of interventional StudiEs on boNe meTastases (PRESENT) cohort; *MRI* magnetic resonance imaging; *SBRT* stereotactic body radiotherapy; *EBRT* external beam radiotherapy; *KPS* Karnofsky performance score

^a^radiculopathy, dermatomal sensory change, and muscle strength of involved extremity is Medical Research Counsil (MRC) 4/5

^b^on a scale from 0 to 10

### Random selection

**Fig. 1:**
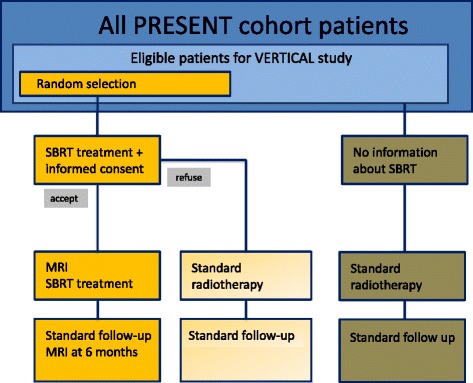
Study design VERTICAL study A large observational cohort of patients with bone metastases is recruited and their outcomes regularly measured (*dark blue* box). Patients within the PRESENT cohort who meet the VERTICAL inclusion criteria are identified as a sub cohort of eligible patients (*light blue* box). Randomly selected patients (*orange* box) are offered the SBRT intervention. The outcomes of these randomly selected patients (i.e. the intervention arm) are then compared with the outcomes of eligible patients not randomly selected who receive standard of care (i.e. the control arm, *brown* boxes)

Eligible patients are randomly selected from the sub cohort on a 1:1 basis with varying block sizes (*n* = six or eight) using an in-house randomization computer program. The radiation oncologist will offer the experimental intervention (i.e. SBRT) to the randomly selected patients. If they accept the treatment offer, they will sign informed consent for participation in the VERTICAL study. Patients who refuse the SBRT will receive care as usual (i.e. standard radiotherapy). According to the cmRCT design, patients in the sub cohort who are not randomly selected will not be informed about the experimental intervention, nor will they be informed about their participation in the control arm of the VERTICAL study. Outcomes in randomly selected patients are compared with the outcomes in eligible patients not randomly selected who received standard radiotherapy (Fig. 1).

### Standard radiotherapy

Standard radiotherapy for symptomatic bony metastases consists of single fraction external beam radiotherapy of 8 Gray (Gy). The radiation oncologist might however choose a multi-fraction regime of 30 Gy in 10 fractions if the patient has a favorable primary tumor (i.e. breast or prostate cancer), a Karnofsky performance score (KPS) of 80–100%, and absence of visceral or brain metastases. The radiation dose distribution usually consists of a single field in posteroanterior direction with the normalization point (100% isodoseline) at 6 cm for a 6 MV photon beam and at 6 or 7 cm for a 10 MV photon beam. The vertebral body should at least receive 80% of the prescribed dose. If necessary, a field in anteroposterior direction is added to the posteroanterior field. Metastases in the cervical spine are usually treated with two lateral opposing fields. The leafs of the multileaf collimator are used to adjust the shape of the treatment field. Prior to treatment, cone beam computed tomography (CBCT) scan images are obtained to verify that the position of the patient is correct with regard to the planning computed tomography (CT). Currently, our department is working on the clinical implementation of auto-planning for single fraction treatment of patients with bone metastases. Automatic treatment plans will then be delivered to the spinal metastases using intensity-modulated radiation therapy (IMRT) technique.

### Stereotactic body radiotherapy

**Fig. 2:**
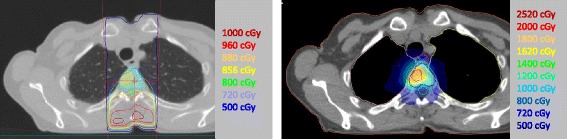
Standard radiotherapy and stereotactic body radiotherapy Comparison of a conventional radiation dose distribution using standard radiotherapy (*left*) with a spinal stereotactic radiotherapy simultaneous integrated boost distribution (*right*) in a patient with a T4 vertebral body metastasis from breast cancer

Patients in the experimental arm undergoing SBRT are immobilized with an S-frame thermoplastic mask in case of skull or cervical spine tumors extending to the upper thoracic (T3) vertebral body. In case of lower thoracic and lumbar lesions, and rib and pelvic lesions, they are immobilized using a vacuum mattress (BlueBAG™, Elekta, Stockholm, Sweden). Magnetic resonance imaging (MRI) is used to delineate the gross tumor volume (GTV), clinical target volume (CTV), and the organs at risk (OAR). We use MRI guidance to deliver stereotactic radiotherapy to the visible metastasis (i.e. GTV) exclusively. With the aid of T1 weighted, T2 weighted, and diffusion weighted imaging (DWI) sequences, it is possible to delineate the GTV accurately [15, 16]. Adjacent normal appearing bone may harbor subclinical disease and could potentially serve as a source for a local recurrence [17]. Therefore, the bony compartment containing the GTV (i.e. the CTV, which consists of the entire vertebral body, pedicle, transverse process, lamina, or spinous process) is prescribed 8 Gy in order to treat subclinical disease whereas the metastasis receives 18 Gy (Fig. 2). This *simultaneous integrated boost* approach has the potential advantage of lowering the risk of vertebral compression fractures by sparing the unaffected, healthy bone tissue surrounding the metastasis while also treating subclinical disease. When necessary, an equivalent dose may be given using another fractionation schedule: 30 Gy in three fractions to the visible metastasis with 15 Gy in three fractions to the bony compartment or 35 Gy in five fractions with 20 Gy in five fractions to the bony compartment. Possible reasons to fractionate the dose might be proximity of visible metastasis to the spinal cord or more than two consecutive spine segments involved. Treatment planning is performed on pre-treatment CT and MRI scans that are co-registered to yield information on all relevant structures for assessing dose distribution. Volumetric modulated arc therapy (VMAT) treatment plans are generated for SBRT patients. Dose constraints are set for the OAR based on institution specific guidelines. These constraints, and particularly the constraint of the spinal cord, are of primary concern. If necessary, dose deliverance to the GTV will be limited in order to meet these constraints [18]. For all patients, an online CBCT scan is acquired with the patient in treatment position on the treatment couch just before start of the irradiation. The CBCT scan yields the exact position of the bony anatomy and is registered to the pre-treatment CT and MRI data. The alignment of the patient, or more specifically the affected vertebra bodies, on the CBCT scan is compared with the pre-treatment CT and MRI scans. After possible correction a second CBCT is performed between the two VMAT arcs. A third CBCT is taken post-treatment to document stability of the target during treatment.

### Primary endpoint

Primary endpoint of this study is complete or partial pain response at three months. Pain response is defined according to the International Bone Metastases Consensus Endpoints for Clinical Trials (Table 2) [19]. A pain score of zero with no concomitant increase in analgesic intake compared to baseline is defined as complete response. Partial response is pain reduction of at least two points on a scale of 0–10 without increase in analgesic intake and/or analgesic reduction of at least 25% from baseline without an increase in pain. Pain progression is defined as an increase in pain score of at least two points above baseline with stable analgesic use and/or as an increase of at least 25% in analgesic use compared to baseline with at least stable pain scores. All responses not captured with complete and partial response or pain progression are considered indeterminate response. Pain is measured by the Brief Pain Inventory (BPI), which has been validated for use in advanced cancer patients to assess pain and functional interference stemming from bone metastases [20].

**Table 2:** Response rate to radiotherapy according to the international consensus [19]

Responders	
Complete response	Pain score of 0 and stable or reduced OMED
Partial response	Pain reduction of 2 points on a 0–10 scale or more and/or OMED reduction by 25% or more
Non-responders	
Pain progression	Increase of 2 points on a 0–10 scale or more above baseline, and/or OMED increased by 25% or more
Indeterminate response	Any response including stable disease that is not captured by complete or partial response or pain progression

*OMED* daily oral morphine equivalent

### Secondary endpoints

Secondary endpoints include local tumor control, duration of pain response, toxicity, vertebral compression fractures, QOL, and overall survival. Evaluation of local tumor control will be based on imaging acquired during follow-up. Duration of pain response starts at response until pain progression or end of follow-up using information provided by the BPI. A radiation oncologist records toxicity according to the National Cancer Institute’s Common Terminology Criteria for Adverse Events, version 4.0 [21] six weeks after radiation treatment. Toxicity occurring after 6 weeks, (serious) adverse events (SAEs), and hospitalization are registered in the context of the PRESENT cohort. Information about toxicity is based on clinical follow-up data and biannual patient-administered questionnaires on health status and hospitalization. All patients in the SBRT arm undergo an additional MRI scan six months after radiation in order to assess vertebral compression fractures. Since most compression fractures occur four months after radiation treatment [22], this 6-month-MRI captures most incidents. In case of clinical suspicion of a vertebral compression fracture, obtaining the MRI scan will be advanced as deemed appropriate. Quality of life is measured by the EORTC QLQ-C15-PAL general questionnaire [23] and the bone metastases-specific module, the EORTC QLQ-BM22 [24]. The EORTC QLQ-C15-PAL is an abbreviated 15-item version of the EORTC QLQ-C30 specially developed for use in palliative care. In order to evaluate the cost-effectiveness, patients are also provided with the EQ-5D questionnaire. Patients fill out these QOL questionnaires and the BPI before the start of radiation treatment (baseline) and after one, two, three, and six months, and every six months thereafter. The BPI is provided after two and six weeks as well. We make use of the digital patient tracking system PROFILES, so patients are able to complete the questionnaires online after secured login [25]. Overall survival is monitored within the PRESENT cohort by clinical follow-up and via an electronic link with the Municipal Personal Records Database.

### Safety

We will report treatment induced SAEs within 15 days following notification through a government based internet portal to the accredited institutional review board that approved the protocol. Treatment induced SAEs that result in death or are life threatening will be reported within seven days.

### Sample size considerations

Based on the most recent meta-analysis, we expect a pain response in 60% of patients following standard radiotherapy [3]. Pain response after stereotactic radiotherapy is assumed to be 85% [8, 9]. We expect that approximately 90% of patients who are offered SBRT treatment, will accept the offer. Cross-over from control arm to the SBRT treatment arm is extremely unlikely, since only patients who are randomly selected to receive SBRT are informed about the treatment. Taking a one-sided α of 5% and a power of 80%, we require 49 patients per treatment arm to show a statistically significant difference of 15% in pain response. The reason to choose a one sided α is that, although improbable, inferior pain response after stereotactic treatment would lead to the same action as no difference at all between the two treatment regimen. This is because the SBRT treatment will only be implemented if it is significantly better than the usual care, since SBRT treatment is more complex, less convenient for patients, and more expensive than standard radiotherapy. Finally, to allow for a 10% drop out rate, recruitment of 55 patients per group is intended. We expect to complete recruitment within 18 months based on the number of patients we treat in our center annually.

### Data analysis

Data will be analyzed according to the intention to treat principle. Data of eligible patients who were randomly offered stereotactic radiotherapy will be compared with eligible patients who were not randomly selected and received standard radiotherapy. In case of dropout (i.e. patients not surviving longer than three months or patients unable to provide pain scores and analgesic use), a worst-case analysis will be performed: dropped-out patients will be classified as non-responders. In case of substantial refusal of the SBRT offer in the intervention arm, instrumental variable analysis will be used to account for non-compliance [26]. The primary outcome (i.e. proportion of patients with response to radiotherapy) will be presented in absolute numbers and proportions. Differences in pain response will be compared by χ2 test. If randomization fails, imbalances between baseline characteristics will be adjusted by logistic regression analysis. Differences in duration of response and overall survival will be analysed by Kaplan-Meier analysis and log rank test. Toxicity will be presented as the overall incidence of grade 3–4 toxicity and incidence of vertebral compression fractures. Differences will be tested with the χ2 test. A comparison in QOL will be made between the baseline QOL and at predefined intervals after treatment. A change of 10% of the scale breadth will be considered a clinically relevant change of QOL [27]. Data will be presented as improved (≥10% increase), stable, or worsened (≥10% decrease) QOL. We will evaluate the pattern of QOL as a continuous outcome over time using mixed models. Differences with a *P*-value <0.05 will be considered statistically significant. We have planned to perform an interim analysis after inclusion of half of the patients (i.e. 55 patients) when they have completed their follow up (i.e. three months pain assessment).

## Discussion

In this report, we present the rationale and design of the VERTICAL trial. In this randomized study, we investigate whether SBRT can increase the proportion of patients with (complete or partial) pain response. Although standard radiotherapy is moderately effective in achieving pain relief in most patients with spinal metastases, up to 40% of patients do not experience any pain relief and complete response occurs in only 30% of responders [3]. Presently, it is not exactly understood why some patients do not respond (adequately) to standard radiotherapy. A factor that may play a role in the suboptimal response to standard radiotherapy is the way the radiation dose is delivered. Barton and colleagues [28] showed that the dose received by the vertebral column using standard radiation techniques varies by up to 50%. For instance, when using a direct posteroanterior field to deliver 8 Gy at a depth of 5 cm, metastases located in deep vertebrae receive less than 50% of the prescribed dose. This is important, since 4 Gy in one fractions is proven to be less effective than 8 Gy [29–31]. If there is indeed a threshold dose below which pain relief is less likely and of slower onset, it may be important to ensure that the vertebral metastasis receives the dose intended. However, the low tolerance of the spinal cord to radiation limits the standard radiation dose to a level that below the optimal therapeutic dose thus providing a less than optimal response. Precise confinement of the radiation dose, even including dose escalation in addition, should increase the probability of pain relief while the risk of injury to the spinal cord is minimized. Several retrospective and prospective phase II studies have indeed shown the safety and efficacy of SBRT in spinal metastases [5, 6].

Most studies on spinal SBRT included a heterogeneous patient population, including previously unirradiated patients, patients who needed reirradiation, and post-operative SBRT, and these categories include patients with or without solitary spine metastases [8, 32]. We include all unirradiated patients with spinal metastases including patients with diffuse metastases, and mild neurological complaints. In this way, we deliberately chose a pragmatic approach since we expect that this would be the patient population that is going to be treated once the benefits of SBRT would have been established. In order to investigate the effect of SBRT without the effect of additional treatments, we however exclude patients who received previous standard or stereotactic body radiotherapy or surgery to the index site. As pragmatic trials investigate the effectiveness of medical treatment strategies under usual conditions, the standard strategy (i.e. 8 Gy in a single fraction, or for selected patients 30 Gy in 10 fractions) will be compared to the SBRT strategy (which includes more dose schedules). Still, the biological effective dose (BED) of the three dose regimen is much higher compared to the BED of the conventional dose regimen. If there is a difference in pain response after SBRT compared to standard radiotherapy, we should be able to detect that differences despite the use of multiple radiation dose schedules. Traditionally, stereotactic radiotherapy in metastatic bone disease is intended for patients with spinal metastases. However, SBRT is increasing being applied in the treatment of non-spine osseous metastases [33]. Since spinal metastases are similar to non-spine osseous metastases in terms of bone involvement and pain relief after standard radiotherapy [34, 35], the response after SBRT in spinal and non-spine osseous metastases is likely to be similar as well. Therefore, we have extended the VERTICAL inclusion criteria to patients with non-spinal bony metastatic disease.

To our knowledge, six other randomized studies on spinal SBRT are currently being conducted (Table 3) [36–41]. Only two other trials require both CT and MRI imaging for the delineation of the spinal metastases [37, 38], however, these trials delineate the whole bony compartment (i.e. the CTV) that contains the metastasis instead of using an simultaneous integrated boost approach. They also have strict instructions on how to apply the standard and stereotactic body radiotherapy in contrast to our more pragmatic approach, offering radiation oncologists leeway in fractionation schedule. Furthermore, the VERTICAL trial distinguishes itself from these trials by applying the cmRCT design. The cmRCT design was proposed as a variant of classic pragmatic randomized controlled trials (RCTs) and addresses some common difficulties associated with those RCTs, such as disappointment bias, drop-outs, slow recruitment, and poor generalizability [13]. Patients and doctors often have a strong preference for the experimental treatment that has not proven to, but is expected to be superior. Investigators of the RTOG 0631 trial indeed experience that patients and their physicians prefer the SBRT treatment over standard radiotherapy [Samuel Ryu, personal communication]. Consequently, patients allocated to the standard arm may show disappointment when reporting outcomes. This is of particular concern since the primary endpoint consists of a subjective outcome (i.e. pain scores). By using the cmRCT design however, control patients are unaware of being allocated to the control arm, which will prevent disappointment bias in observed outcomes. Furthermore, standard of care is likely to be unaffected by treatment allocation and will therefore better resemble routine practice. We also expect lower drop-outs rates since patients in the control arm are not likely to withdraw from standard care, which may be of particular interest in this fragile patient population. Because of this fragility, researchers in this field should make an effort to optimize recruitment rates. The use of the cmRCT design may foster recruitment rates by its unique informed consent procedure. A reason not to take part in classic randomized studies might be that patients cannot be guaranteed to receive the desired experimental treatment. Furthermore, once participating, patients are often allowed to participate in one trial at a time only. By contrast, patients participating in a cmRCT study give broad informed consent to participate in randomized trials, but not to specific trials which may increase recruitment rates. Moreover, the cmRCT cohort offers an infrastructure which allows the conduct of randomized trials simultaneously. Finally, recruitment in cohort studies is usually more manageable compared with recruitment in RCTs. The inclusion rates in the PRESENT cohort for example are promising: the participation rate is 83%, and 88% of the participating patients have given informed consent for broad randomization to experimental interventions. The use of a cohort in cmRCT studies offers more potential advantages. Palliative patients willing to participate in randomized trials often represent a relatively healthier and higher-educated subgroup. By using a cohort as a recruitment pool for RCTs, a more routine population is included since recruitment for cohort studies is generally less selective. Moreover, the cohort provides information on baseline characteristics and outcome measurements (i.e. the regular cohort measures) of drop-outs, which is essential in the data analysis. Patients allocated to the control arm, are cohort participants who receive the current standard of care (i.e. standard radiotherapy in the PRESENT case). In our department, the standard of care for patients with bone metastases will change from standard radiotherapy to automatically generated conformal treatment plans. Would the VERTICAL trial have been conventionally conducted, this could have been problematic since control patients in the VERTICAL trial would then have been withhold from standard of care. However, the cmRCT design has the advantage that experimental interventions are compared with the most up-to-date standard of care, instead of competing with outdated treatments, which is often the case in completed classic RCTs. Finally, a valuable feature of the cmRCT design is the opportunity to evaluate and quantify the acceptance rates of the offered treatment (i.e. SBRT). This offers new insights into patient preferences and reasons for refusal of SBRT. We feel that prevention of disappointment bias, more efficient and less selective patient recruitment, up-to-date standard of care, and quantifying patients’ preference could significantly improve trials conducted according to the cmRCT design.

**Table 3:** Randomized trials on SBRT for spinal metastases^a^

Name, institution	Start date, sample size	Patients	SBRT treatment	Comparator	Primary Endpoint
Mahadevan et al. [36] Beth Israel Deaconess MC	01–2012 81	Number of sites not stated; Pain ≥ 5; No rapid neurologic decline	Total dose unknown in 1, 3, or 5 fractions; No more information provided	Standard EBRT in 10 fractions	Pain response^b^
RACOST [37] Radboud UMC Nijmegen	06–2015 382	Number of sites not stated; May have other visceral metastases; Pain ≥ 5; No neurologic deficit	Any modern system; 20 Gy in one fraction; Delineation with MRI and CT; Target volume is GTV, with bony CTV expansion, PTV margin ≤ 3 mm	Standard EBRT single dose of 8 Gy, no restrictions to radiation technique	Pain response taking administration of opioids into account^b^
RTOG 0631 [38] Henry Ford Hospital	11–2011 395	Up to 3 spinal sites; May have other visceral metastases; Pain ≥ 5; No rapid neurologic decline	IMRT or other dose painting technique; 16 or 18 Gy in one fraction; Delineation with MRI and CT; Target volume is involved VB	Standaard EBRT single dose of 8 Gy, 2D and 3D conformal therapy	Pain response (increase or decrease of ≥ 3 points) at 3 months
SMART [39] Heidelberg University	12–2014 60	Up to 2 spinal sites; No neurologic deficit	IMRT; 24 Gy in one fraction; Delineation with CT; Target volume is involved VB with PTV margin	Standard EBRT 30 Gy in 10 fractions, 3D conformal planning	Pain response (increase or decrease of > 2 points) at 3 months
SPIN-MET [40] University of Erlangen-Nürnberg	03–2013 155	Number of sites not stated; May have other visceral metastases; No rapid neurologic decline	36 Gy in 12 fractions plus integrated boost 48 Gy in 12 fractions; No more information provided	Conventional EBRT 30 Gy in 10 fractions	Tumor control defined as time to progression on MRI
Tingting et al. [41] Cancer Hospital of Shantou UMC	03–2014 100	Up to 3 spinal sites	24 Gy in 2 fractions; No more information provided	Conventional EBRT 30 Gy in 10 fractions	Pain response taking administration of opioid into account^b^
VERTICAL University Medical Center Utrecht	01–2015 110	Up to 2 spinal sites; May have other visceral metastases; Pain ≥ 3; no rapid neurologic decline	VMAT; 18 Gy in one fraction or fractionated equivalent; Delineation with MRI and CT; Target volume with simultaneous integrated boost	Standard of care for standard radiotherapy	Pain response (increase or decrease of ≥ 2 points) taking administration of opioid into account at 3 months

*CT* computed tomography, *CTV* clinical target volume, *EBRT* external beam radiotherapy, *IMRT* image guided radiotherapy, *GTV* gross tumor volume, *MC* medical center, *MRI* magnetic resonance imaging, *PTV* planning target volume, *VB* vertebral body

^a^Excluding studies on oligometastases including spinal oligometastatic disease, comparing surgery with SBRT, and studies including non-spinal lesions as well

^b^Time point at which endpoint is measured not given

In conclusion, the VERTICAL study is a pragmatic randomized trial, following the cmRCT design, which compares stereotactic radiotherapy with standard radiotherapy in patients with spinal metastases in terms of pain response, with the ultimate goal to improve quality of life.

## Abbreviations

BED: Biological effective dose
BPI: Brief pain inventory
CBCT: Cone beam computed tomography
cmRCT: Cohort multiple randomized controlled trial design
CT: Computed tomography
CTV: Clinical tumor volume
EORTC: European organization for research and treatment of cancer
GTV: Gross tumor volume
Gy: Gray
IMRT: Intensity-modulated radiation therapy
KPS: Karnofsky performance score
MRI: Magnetic resonance imaging
MV: Megavoltage
OAR: Organs at risk
PRESENT: Prospective evaluation of interventional StudiEs on boNe meTastases cohort
PROFILES: Patient reported outcomes following initial treatment and long term evaluation of survivorship
PROMs: Patient-reported outcomes
QOL: Quality of life
RCT: Randomized controlled trial
SAE: Serious adverse event
SBRT: Stereotactic body radiotherapy
VERTICAL: Comparison of conVEntional RadioTherapy with stereotactIC body radiotherapy in patients with spinAL metastases
VMAT: Volumetric modulated arc therapy
